# Improving diabetes control for Syrian refugees in Jordan: a longitudinal cohort study comparing the effects of cash transfers and health education interventions

**DOI:** 10.1186/s13031-021-00380-7

**Published:** 2021-05-25

**Authors:** By Emily Lyles, Stephen Chua, Yasmeen Barham, Kayla Pfieffer-Mundt, Paul Spiegel, Ann Burton, Shannon Doocy

**Affiliations:** 1 Department of International Health, Johns Hopkins Bloomberg School of Public Health, 615 N. Wolfe Street Suite E8132, Baltimore, MD 21205 USA; 2 Medair, Amman, Jordan; 3 United Nations High Commissioner for Refugees, Geneva, Switzerland

**Keywords:** Cash transfers, Conditional cash, Diabetes, Humanitarian assistance, Syrian refugees, Jordan

## Abstract

**Background:**

Cash transfers are an increasingly common intervention in the Syrian refugee response to meet basic needs, though there is little known of their potential secondary impact on health outcomes in humanitarian settings.

**Methods:**

A quasi-experimental prospective cohort study was implemented from October 2018 through January 2020 to assess the effectiveness of multi-purpose cash (MPC), community health volunteer (CHV)-led education, combined with conditional cash transfers (CCT) with respect to health measures among Syrian refugees with type II diabetes in Jordan.

**Results:**

CHV + CCT participants had the highest expenditures at endline and were the only group with statistically significant increases in payments for outpatient diabetes care (25.3%, *P* < 0.001) and monthly medication costs (13.6%, P < 0.001). Conversely, monthly spending on diabetes medication decreased significantly in the CHV only group (− 18.7%, *P* = 0.001) yet increased in the MPC and CHV + CCT groups. Expenditures on glucose monitoring increased in all groups but significantly more in the CHV + CCT group (39.2%, *P* < 0.001).

The proportion of participants reporting regular diabetes care visits increased significantly only in the CHV + CCT group (15.1%, *P* = 0.002). Specialist visits also increased among CHV + CCT participants (16.8%, *P* = 0.001), but decreased in CHV only participants (− 27.8%, *P* < 0.001). Decreases in cost-motivated provider selection (− 22.8%, *P* < 0.001) and not receiving all needed care because of cost (− 26.2%, P < 0.001) were significant only in the CHV + CCT group.

A small significant decrease in BMI was observed in the CHV + CCT group (− 1.0, *P* = 0.005). Decreases in HbA1C were significant in all groups with magnitudes ranging from − 0.2 to − 0.7%. The proportion of CHV + CCT participants with normal blood pressure increased significantly from baseline to endline by 11.3% (*P* = 0.007).

**Conclusions:**

Combined conditional cash and health education were effective in improving expenditures, health service utilization, medication adherence, blood pressure, and diabetes control. The lower cost health education intervention was similarly effective in improving diabetes control, whereas unconditional cash transfers alone were least effective. Study findings suggest that conditional cash or combined cash and health education are promising strategies to support diabetes control among refugees and that where the purpose of MPC is to improve health outcomes, this alone is insufficient to achieve improvements in the health of refugees with diabetes.

**Supplementary Information:**

The online version contains supplementary material available at 10.1186/s13031-021-00380-7.

## Introduction

Displacement is increasingly occurring in urban and middle-income settings and the profile of displaced populations has evolved, becoming older and having an epidemiological profile marked by high prevalence of chronic noncommunicable diseases (NCDs) [1–3]. With 5.5 million refugees, more than any other country, and a comparatively older population, the Syria crisis has accelerated this shift [4]. The high prevalence of NCDs in many refugee populations presents unique challenges to host country health systems in providing appropriate continuity of care and access to medications. Inadequate treatment for NCDs, particularly for diabetes, can lead to complications necessitating sophisticated, often costly, treatments and preventable adverse health outcomes [5–7]. Hypertension and diabetes are the most common NCDs among Syrian refugees in Jordan with adult prevalence estimated at 10.7 and 6.1%, respectively, in 2014 [8, 9].

Over 650,000 Syrian refugees are registered in Jordan alone, more than 80% of whom are settled in Jordanian communities outside of camps [10]. Health assistance for urban Syrian refugees in Jordan is provided through public sector facilities at reduced rates based on Ministry of Health (MoH) policies. Healthcare was initially provided free of charge for Syrian refugees registered with the United Nations High Commissioner for Refugees (UNHCR) until 2014 when out-of-pocket payments increased, requiring Syrian refugees to pay the same rates as uninsured Jordanians. In 2018, policy revisions required refugees to pay 80% of foreigner rates for care at MoH facilities, which again increased out-of-pocket payments [11]. Since April 2019, Jordan has reverted to the 2014 policy such that Syrian refugees again pay the same rates as uninsured Jordanians; however, health service utilization for NCDs notably declined among Syrian refugees after the initial 2014 user fee increase, suggesting the cost of NCD care, even at reduced amounts, is a widespread challenge [12]. Some refugees have been able to access free or very low cost NCD care at facilities operated by non-governmental organizations (NGOs); others also chose to seek care in the private sector, thus, there is a diversity with respect to sector and out-of-pocket expenditures for care.

While health education has been studied extensively in higher-income stable contexts, an increasing body of literature has also demonstrated the benefits and utility of health education for improving chronic disease care in humanitarian settings [13, 14]. This is particularly true when education is combined with community-based routine monitoring and individual counseling. Treatment support such as community-based clinical, psychosocial, and educational support to patients and their families have proven to be acceptable and effective means of improving treatment adherence for those with diabetes, hypertension, and several other chronic conditions in humanitarian and development contexts [15–18]. Support to host country health systems to build capacity to address refugee health needs is critical but cannot always address the issue of health care affordability. Cash transfers have the potential to fill this gap and fundamentally change approaches to providing humanitarian assistance. While cash transfers are purported to be more effective and efficient than in-kind assistance to improve local economies, and to provide more choice and dignity for beneficiaries, there is little evidence as to how cash transfers affect health in humanitarian crises [19–21]. A 2015 systematic review of the effect of unconditional cash transfers on use of health services and health outcomes in humanitarian emergencies in low- and middle-income countries identified only three appropriate studies, all of low quality and focused on drought contexts [19]. The review concluded that there is a need for higher quality evidence from varied contexts to determine the effectiveness of cash transfers for improving utilization of health service and health outcomes in humanitarian settings.

Cash transfers are an increasingly common modality in humanitarian response. In 2019, approximately US$5.6 billion in global humanitarian assistance was provided through cash and voucher assistance (CVA), a twofold increase from 2015 [22]. In Jordan alone, nearly 40% of the US$10.4 million allocated through the Jordan Humanitarian Fund in 2018 was provided as cash assistance [23]. UNHCR and the World Food Programme (WFP) currently provide the largest cash assistance programs in Jordan, both of which are intended to help households meet basic needs, based on the calculation of a minimum expenditure basket. UNHCR provides eligible households with monthly unrestricted cash transfers, or multipurpose cash (MPC), valued between 80 and 155 JOD (US$113–219) based upon household size and vulnerabilities [24]. WFP provides cash transfers to vulnerable refugee households through their “Choice” program, wherein 15–23 JOD (US$21–32) per household member can be redeemed monthly either by withdrawing cash from automated teller machines (ATMs) or by using the card to purchase food items at one of WFP’s 200 partner shops [25].

MPC may be used by households as they deem fit. Receipt of cash may be conditional upon a variety of desired behaviors such as completion of vaccination, attending medical appointments, or participation in health education sessions. Despite extensive use of cash transfers in the Syrian refugee response, studies assessing their effectiveness for improving primary or secondary health outcomes are limited, in particular for conditional cash transfers (CCTs) [26–28]. Frequent debates among humanitarian practitioners include whether conditionality has an impact on outcomes, for example by restricting how funds can be used, and if cash alone is sufficient or if additional supports (e.g., education) are needed to realize change. In the absence of well-designed research that assesses the effectiveness of cash transfers on health service utilization and health outcomes in humanitarian settings, this study assesses the effect of CCTs, MPCs, and health education separately and in combination on health-seeking behavior, service utilization, and disease control among Syrian refugees with diabetes in Jordan to inform health sector cash transfer program design for both current and future humanitarian responses.

## Methods

A quasi-experimental prospective cohort study was implemented from October 2018 through January 2020 to assess the effectiveness of CCTs, MPCs, and health education for improving health measures among Syrian refugees with type II diabetes residing outside of camps in Amman and Zarqa governorates of Jordan. Syrian refugees with a prior diagnosis of type II diabetes in households classified as vulnerable (Vulnerability Assessment Framework (VAF) score of three or four) were followed for 1 year and received one of the following interventions:

Community health volunteer (CHV) intervention (“CHV” group): CHV group participants attended quarterly CHV-led group health education meetings focused on lifestyle behaviors, health-seeking behavior/service utilization, and medication adherence. In addition, CHVs conducted quarterly home visits to reinforce education meeting content; assess participants’ height, weight, and blood pressure; inform participants of their risk level; and provide individualized counseling on recommended behavioral modifications and care-seeking. Informational brochures on diabetes education topics were also distributed during the meetings and home visits.

CHV intervention + conditional cash transfer intervention (“CHV + CCT” group): CHV + CCT group participants received the same quarterly group education sessions and home visits described in the CHV intervention. In addition, they received CCTs valued at 150 JOD (US$211) on a quarterly basis; the transfer amount was determined based on the average costs of diabetes medication and care-seeking at Ministry of Health facilities; it should however be noted that free care is available from some NGOs and that many refugees also opt seek care in the private sector. Continued receipt of transfers was conditional upon evidence of transfer expenditures; transfer spending was verified directly by the study team at quarterly education sessions during which participants were required to provide receipts for appropriate services from health facilities and/or prescription fill verification.

Multi-purpose cash transfer intervention (“MPC” group): Individuals identified by UNHCR at the start of the study or during screening as MPC beneficiaries were also included. Individuals in this group benefited from UNHCR’s household level MPC, which ranged in value from 80 to 155 JOD (US$113–219) depending on household size and were provided monthly. Individuals in this group received no further intervention other than being informed of blood glucose level and blood pressure, which were measured during enrollment and endline data collection for study participants in all three study groups. Though not initially planned for the study, the opportunity to include this third group was leveraged to compare multiple modalities of assistance; however, for budgetary reasons, the MPC group did not receive the CHV education intervention.

Sample calculations were based on the proportion of individuals with “controlled” diabetes and were informed by unpublished data collected by the research team in a previous study of NCD care-seekers in Lebanon, which observed a baseline control level of 46% for type II diabetes patients. Estimates for other dichotomous measures assume the most conservative prevalence rate of 50% for sample size calculations, ensuring the ability to detect statistically significant differences of the same magnitude from all other prevalence rates. Assuming power = 0.80 and ≤ 14% loss to follow-up, a minimum sample of 600 individuals (200 per group) was determined to be sufficient to detect statistically significant differences ≥15% in dichotomous outcomes. Sample sizes could not be accurately calculated for continuous measures (glycated hemoglobin [HbA1C], body mass index [BMI], etc.) because distributions in the Syrian refugee population are unknown.

Study areas were selected based on availability of free or highly subsidized NCD care, low health service accessibility, and high refugee caseload [6]. Within Amman and Zarqa governorates, districts with sufficiently large numbers of eligible refugees were selected for the study and divided into two groups with similar numbers of refugees with diabetes identified during screening as eligible for participation and not receiving MPC. One group of selected districts was randomly assigned to the CHV group and another to the CHV + CCT group. Refugees with diabetes receiving UNHCR MPC were enrolled proportionally across study districts.

Individuals age 18 through 70 years were eligible to participate if they were a member of a vulnerable refugee household (VAF score of three or four) registered with UNHCR as a non-camp household in Amman or Zarqa governorate and had a prior type II diabetes diagnosis supported by evidence such as health records, prescription(s), and/or laboratory result(s). Individuals were deemed ineligible if they had a prior cancer diagnosis, were insulin dependent, on dialysis, pregnant, or were not mentally competent to consent to participation. For the MPC group, participants’ households must have been beneficiaries at enrollment, whereas participants in the CCT and CHV groups must not have been receiving UNHCR MPC.

Potential participants were identified using UNHCR refugee registration lists, which provided information on household location, vulnerability level, and whether member(s) have a chronic health condition. Households were called to identify the household member(s) with diabetes, and to verify study eligibility and residence location; list-based screening continued for selected geographic areas until lists were exhausted. Individuals confirmed to meet eligibility requirements in the screening phone call then received a baseline home visit for confirmation. In cases where two or more eligible individuals were identified within a household, both individuals were enrolled. Participants were enrolled from October 2018 through January 2019 and received interventions for 1 year.

Oral informed consent was obtained by CHVs who received training in human subjects’ research, informed consent processes, appropriate data collection practices, study data collection tools, diabetes and hypertension management/care, and health education. Consent was obtained from all individuals in the initial screening call and again during home visits before confirming eligibility and enrollment. Participants were also read an abbreviated consent statement prior to subsequent interviews and were provided the opportunity to ask questions and decline to continue participation. Quantitative data collection consisted of baseline and endline interviews lasting approximately 1.5 h that focused on demographic and health characteristics and outcome measures.

Primary outcome measures are provided in detail in Additional File 1 and included health expenditures, health service utilization, and health outcomes. Health expenditure measures consisted of both the proportion of participants reporting any expenditures and average expenditures amounts for (1) health facility payments for most recent outpatient diabetes care, (2) monthly medication costs (for all medications and only those for diabetes), and (3) blood glucose monitoring supplies, as well as routine spending on health, including asset sales and borrowing money to pay for health. Diabetes care utilization measures included the proportions of participants receiving regular diabetes care, unable to receive regular care due to cost, and reporting one or more visits to select types of providers. Additional care utilization measures focused on the most recent care visit (within the past year) were also assessed, including the type of visit and sector utilized, as well as the proportion of participants who selected providers due to cost and those who did not receive all needed care because of cost. Health outcomes included medication adherence, blood sugar and foot checking, smoking status, diet, and physical activity measures as well as clinical outcome measures such as BMI, blood pressure, and HbA1C. Diabetes control was defined as having HbA1C < 7.0%.

Data analysis was performed using Stata 13 (College Station, TX). Descriptive differences between intervention groups (i.e., MPC vs. CHV vs. CHV + CCT) were examined using chi-square and t-test methods for binary/categorical and continuous variables, respectively. Regression models were used to evaluate change in adherence and monitoring, lifestyle risk factors, health service utilization, health expenditures, and clinical measurements from baseline to endline in each group, both unadjusted and controlling for differences in participant characteristics. Differences in binary outcomes between intervention groups were estimated using linear probability models with main terms for study group, time (i.e., baseline/endline), and interactions between intervention group and time. To estimate differences in continuous outcomes, analogous log-linear models were used; log transformation was required for health expenditure outcomes due to their skewed distribution. All models utilized cluster-robust standard errors with clustering defined at the individual level, allowing for correlation between observations for each participant. Financial indicators are presented in U.S. Dollars (US$) using an exchange rate of 1.41 JOD/US$1 [29]. Monetary variables were assessed for outliers using visual examination and investigation of points falling three or more standard deviations from the mean. Outliers were checked with field teams and corrected where possible; remaining outliers were Winsorized to three standard deviations from the mean.

This study was approved by the Institutional Review Board at The Johns Hopkins Bloomberg School of Public Health and the Jordanian Ministry of Planning and International Cooperation.

## Results

### Study population characteristics

**Fig. 1:**
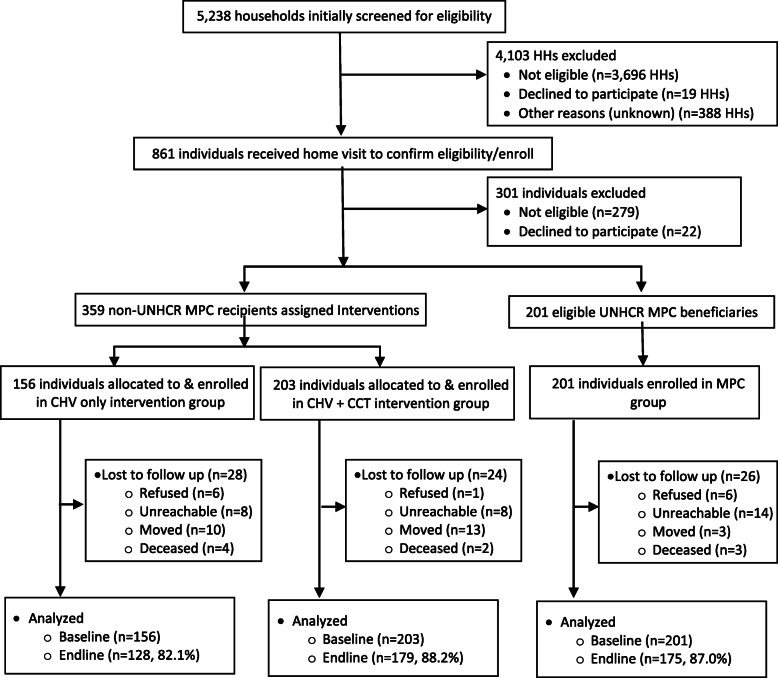
Study Profile Flow Diagram

A total of 560 individuals were enrolled in the study including 201 receiving MPC, 156 receiving CHV education only, and 203 receiving CHV education and CCTs. Of those enrolled, 482 (86.1%) were successfully followed for 1 year to complete endline interviews; reasons for incomplete/loss to follow-up included refusal to continue study participation (*n* = 13), moving within or outside of Jordan (*n* = 26), death [unrelated to study participation] (*n* = 9), and inability to reach the participant (i.e., phone number change or work commitments) (*n* = 30) (Fig. 1).

Participant characteristics differed at baseline between the MPC group and the CHV and CHV + CCT groups (Table 1). Compared to CHV and CHV + CCT participants, a significantly larger proportion of MPC participants were female. MPC participants were also significantly older and were more likely to have low educational attainment, be a widow, or live in a household with multiple UNHCR registration cases.^1^ MPC participants also has significantly longer diabetes duration (i.e., diagnosed longer ago) than those in the CHV and CHV + CCT groups. Household economic characteristics (Table 2) also differed among intervention groups, with CHV group participants reporting significantly higher incomes at both baseline and endline and CHV + CCT group participants reporting significantly higher expenditures at endline. While only those in the MPC group received MPC from UNHCR at baseline, 83.4% of participants in the MPC group reported receiving UNHCR MPC at endline, as did 32.8% of the CHV group and 17.3% of the CHV + CCT group (*P* < 0.001). Significantly more MPC participants also reported receiving WFP CVA at baseline relative to other groups (*P* = 0.002); however, similar proportions in all intervention groups reported WFP CVA at endline (*P* = 0.358).

**Table 1:** Baseline Participant Demographic and Clinical Characteristics

	MPC		CHV only		CHV + CCT		*P*-value
	(***N*** = 201)		(***N*** = 156)		(***N*** = 203)		
**Household Demographic Characteristics**							
Household size	6.0	(2.6)	5.5	(2.1)	5.7	(2.4)	0.175
Multiple UNHCR registration cases ^a^	131	(65.2%)	63	(40.4%)	90	(44.3%)	***< 0.001***
**Participant Demographic Characteristics**							
Female sex	137	(68.2%)	86	(55.1%)	107	(52.7%)	**0.004**
Age (years)	56.2	(9.4)	52.1	(8.6)	52.1	(9.2)	***< 0.001***
Highest level of education							
None	61	(30.3%)	29	(18.6%)	29	(14.3%)	**0.007**
Primary school	100	(49.8%)	94	(60.3%)	121	(59.6%)	
Preparatory school	25	(12.4%)	18	(11.5%)	31	(15.3%)	
Secondary school or higher	15	(7.5%)	15	(9.6%)	22	(10.8%)	
Marital status							
Married	144	(71.6%)	130	(83.3%)	168	(82.8%)	**0.031**
Widowed	46	(22.9%)	23	(14.7%)	25	(12.3%)	
Never married / divorced	11	(5.5%)	3	(1.9%)	9	(4.4%)	
**Participant Clinical Characteristics**							
Years since initial diabetes diagnosis	7.5	(6.1)	5.9	(5.2)	5.6	(4.7)	**0.001**
Previously diagnosed comorbidities ^b^							
Any chronic condition(s)	167	(83.1%)	133	(85.3%)	162	(79.8%)	0.388
Hypertension	139	(69.2%)	93	(59.6%)	129	(63.5%)	0.165
Arthritis	59	(29.4%)	36	(23.1%)	47	(23.2%)	0.266
Cardiovascular disease	41	(20.4%)	38	(24.4%)	44	(21.7%)	0.664
Chronic respiratory disease	17	(8.5%)	7	(4.5%)	12	(5.9%)	0.295
Other	79	(39.3%)	73	(46.8%)	74	(36.5%)	0.131

Presented as N (%) for binary/categorical variables and mean (SD) for continuous variables

^a^ Multiple UNHCR registration cases primarily occurred when there was more than one traditionally defined family in a household according to the study’s definition of a household (people who share a living space, meals, and financial resources)

^b^ Each condition as percent of all participants

**Table 2:** Household Economy and Receipt of Humanitarian Assistance at Baseline and Endline (in US$)

		BASELINE							ENDLINE						
		MPC		CHV only		CHV + CCT		***P***-value	MPC		CHV only		CHV + CCT		***P***-value
		(***N*** = 201)		(***N*** = 156)		(***N*** = 203)			(***N*** = 175)		(***N*** = 128)		(***N*** = 179)		
**Income & Expenditures (past month)**															
Income (excluding humanitarian assistance)	Median	282	–	367	–	384	–		212	–	310	–	282	–	
	Mean (SD)	297.9	(215.1)	405.0	(227.0)	383.8	(206.3)	***< 0.001***	232.9	(242.6)	331.0	(278.7)	292.9	(253.9)	**0.005**
Total expenditures	Median	642	–	621	–	660	–		709	–	764	–	852	–	
	Mean (SD)	679.8	(399.6)	685.0	(380.2)	700.8	(395.7)	0.856	790.3	(441.0)	848.4	(480.3)	943.9	(640.7)	**0.025**
**Humanitarian Assistance (past month)**															
**UNHCR MPC**		201	(100%)	0	(0.0%)	0	(0.0%)	–	146	(83.4%)	42	(32.8%)	31	(17.3%)	***< 0.001***
Amount received (per HH)	Median	169	–	–	–	–	–		176	–	169	–	176	–	
	Mean (SD)	174.8	(61.9)	–	–	–	–	–	187.7	(71.6)	175	(56.3)	169.9	(40.3)	0.265
Amount received (per HH member)	Median	31	–	–	–	–	–		31	–	34	–	33	–	
	Mean (SD)	35.9	(21.6)	–	–	–	–	–	39.0	(30.9)	39	(23.3)	36.2	(14.7)	0.871
**WFP Food Assistance**															
Current WFP recipients (%)		197	(98.0%)	142	(91.0%)	182	(89.7%)	**0.002**	167	(95.4%)	117	(91.4%)	168	(93.9%)	0.358
Amount received (per HH)	Median	127	–	106	–	123	–		127	–	106	–	102	–	
	Mean (SD)	137.2	(77.1)	120.5	(66.0)	122.6	(60.3)	**0.043**	138.3	(83.0)	114.0	(66.2)	113.7	(65.1)	**0.003**
Amount received (per HH member)	Median	21	–	21	–	21	–		23	–	21	–	21	–	
	Mean (SD)	23.3	(9.1)	21.8	(7.1)	22.5	(14.5)	0.445	25.3	(18.1)	20.9	(10.3)	21.6	(16.6)	**0.035**
Transfer modality															
E-voucher		9	(4.6%)	14	(9.9%)	15	(8.2%)	0.150	22	(13.2%)	15	(12.8%)	18	(10.7%)	0.765
Choice (e-voucher or cash)		188	(95.4%)	128	(90.1%)	167	(91.8%)		145	(86.8%)	102	(87.2%)	150	(89.3%)	
**Asset Sales and Borrowing**															
Sold assets in past 3 months (%)		50	(24.9%)	43	(27.6%)	59	(29.1%)	0.633	37	(21.1%)	38	(29.7%)	58	(32.4%)	**0.050**
Borrowed money in past 3 months (%)		153	(76.1%)	123	(78.8%)	159	(78.3%)	0.433	133	(76.0%)	92	(71.9%)	125	(69.8%)	0.533
Any current debt (%)		172	(88.7%)	130	(91.5%)	183	(92.0%)	0.485	156	(91.8%)	109	(89.3%)	154	(90.1%)	0.762
Amount of debt ^a^	Median	705	–	765	–	776	–		846	–	846	–	917	–	
	Mean (SD)	931.4	(1024.9)	1431.5	(1778.6)	1481.2	(3172.5)	**0.031**	1619.1	(3184.8)	1994.8	(2603.0)	1491.9	(1929.9)	0.258

Presented as N (%) for binary/categorical variables and median/mean (SD) for continuous variables. Exchange rate: 1 JOD = US$1.41. HH = household

^a^ among those with debt

### Health expenditures

**Fig. 2:**
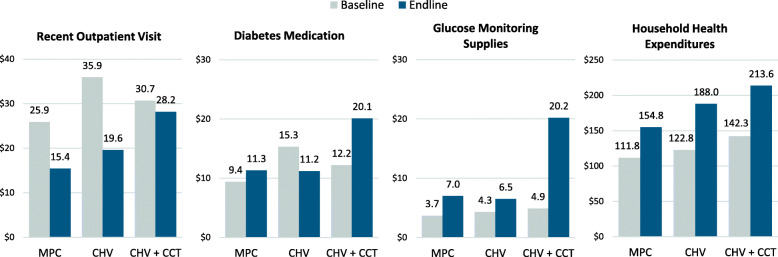
Mean Health Expenditures at Baseline and Endline by Intervention Group (in US$)

Expenditures were assessed for the most recent outpatient diabetes visit, average monthly blood glucose monitoring and medication costs (for all medication and those specifically for diabetes), and for overall household health in the past month. Unadjusted and adjusted change over the study period in each group are presented in Table 3; baseline and endline values and significance tests for each group are provided in Fig. 2 and in Additional File 2.

**Table 3:** Change in Health Expenditures for Diabetes Care and Maintenance (in log US$)

	UNADJUSTED CHANGE							ADJUSTED CHANGE ^**a**^						
	MPC		CHV only		CHV + CCT		*P*-value	MPC		CHV only		CHV + CCT		*P*-value
	Point Est.	(95% CI)	Point Est	(95% CI)	Point Est	(95% CI)		Point Est	(95% CI)	Point Est	(95% CI)	Point Est	(95% CI)	
**Most Recent Health Facility Payments for Outpatient Diabetes Care** ^**b**^														
Any payment at last visit (%)	−4.7%	(−14.2,4.8)	*− 9.8%*	*(− 20.6,1.0)*	***26.8%***	***(18.9,34.7)***	***< 0.001***	−5.8%	(−15.5,3.9)	−9.0%	(− 20.6,2.5)	**25.3%**	**(16.8,33.9)**	***< 0.001***
Total paid at facility for visit ^b^	**−0.5**	**(−1.7,0.7)**	−1.1	(−2.5,0.3)	**1.6**	**(0.7,2.5)**	**0.002**	−0.8	(−2.0,0.4)	−1.1	(−2.6,0.4)	**1.2**	**(0.2,2.2)**	**0.007**
**Average Monthly Medication Costs**														
**Medication for All Conditions**														
Any medication costs (%)	1.4%	(−4.8,7.7)	1.7%	(−5.5,9.0)	***15.7%***	***(9.6,21.8)***	**0.002**	0.3%	(−6.2,6.9)	0.8%	(−7.1,8.7)	***13.6%***	***(7.2,19.9)***	**0.005**
Average monthly medication costs ^c^	0.1	(−0.6,0.9)	0.1	(−0.9,1.0)	***2.3***	***(1.5,3.1)***	***< 0.001***	0.0	(−0.8,0.8)	−0.1	(−1.1,0.9)	***2.0***	***(1.2,2.8)***	***< 0.001***
**Medication for Diabetes**														
Any diabetes medication costs (%)	3.8%	(−4.5,12.1)	**−16.1%**	**(−26.9,-5.3)**	**10.0%**	**(2.2,17.9)**	**0.001**	1.4%	(−7.2,9.9)	**− 18.7%**	**(−30.2,-7.2)**	7.0%	(−1.4,15.3)	**0.001**
Average monthly medication costs ^c^	0.4	(−0.5,1.4)	**−2.1**	**(−3.4,-0.8)**	**1.4**	**(0.5,2.4)**	***< 0.001***	0.1	(−0.9,1.1)	**− 2.4**	**(−3.8,-1.0)**	**1.0**	**(0.0,2.0)**	***< 0.001***
**Blood Glucose Monitoring Supplies** ^**d**^														
Any monitoring supply costs (%)	***15.1%***	***(7.7,22.6)***	**14.9%**	**(5.1,24.8)**	***40.8%***	***(32.2,49.3)***	***< 0.001***	***16.1%***	***(8.4,23.7)***	**13.6%**	**(3.3,23.9)**	***39.2%***	***(30.3,48.1)***	***< 0.001***
Average monthly supply costs ^c^	***1.8***	***(1.0,2.7)***	**1.8**	**(0.6,3.0)**	***5.2***	***(4.1,6.2)***	***< 0.001***	***1.9***	***(1.0,2.9)***	**1.6**	**(0.4,2.9)**	***5.0***	***(3.9,6.1)***	***< 0.001***
**Routine Spending on Health**														
Health Expenditures (past month) ^e^	**−0.6**	**(− 1.1,0.0)**	**0.4**	**(− 0.2,1.0)**	**0.5**	**(0.2,0.7)**	**0.004**	**− 0.7**	**(− 1.3,-0.2)**	0.4	(− 0.3,1.0)	0.2	(− 0.1,0.5)	**0.008**
Sold assets to pay for health ^f^	**−4.7%**	**(−8.8,-0.6)**	− 0.9%	(− 8.4,6.6)	−5.0%	(− 11.2,1.3)	0.656	**−5.2%**	**(− 9.6,-0.8)**	− 1.9%	(− 10.0,6.3)	− 5.4%	(− 11.9,1.1)	0.732
Borrowed to pay for health ^f^	**−11.2%**	**(− 20.6,-1.8)**	**− 12.1%**	**(− 23.2,-1.0)**	**− 12.8%**	**(− 22.4,-3.1)**	0.974	**− 12.5%**	**(− 22.2,-2.7)**	**−14.5%**	**(− 26.2,-2.9)**	**−16.0%**	**(−26.1,-5.9)**	0.876

Exchange rate: 1 JOD = US$1.41. Point Est = point estimate from regression model. Bold italic indicates statistically significant (P < 0.001) findings; bold indicates statistically significant (*P* < 0.05) findings; italic indicates statistically significant (*P* < 0.10) findings. *P*-values are for three group comparison of magnitude of change during the study period

^a^ Adjusted analyses controlled for participant sex, age, education level, and marital status; household size and the presence of more than one UNHCR registration case; prior diagnoses of hypertension, other chronic NCD diagnoses, and years since initial diabetes diagnosis; total household expenditure in the prior month; and receipt of humanitarian assistance, specifically the total value of cash assistance received in the prior month, and current WFP beneficiary status/transfer modality (voucher or Choice); ^b^ includes consultation fees, diagnostic testing, and medications obtained at health facility during the most recent visit to health facility, hospital outpatient department, or emergency room (without overnight stay); ^c^ among all participants; ^d^ at home or in a pharmacy; ^e^ Includes expenditures for consultation, diagnostic testing, medication, and associated transportation; ^f^ in the past three months

Individual health expenditures were generally similar across groups at baseline; however, at endline participants in the CHV + CCT group reported expenditures significantly more often and in higher amounts compared to CHV only and MPC participants (Fig. 2). With respect to payment for the most recent outpatient diabetes care visit, the adjusted proportion of participants with payments increased significantly among CHV + CCT participants (25.3%, CI: 16.8,33.9%; *P* < 0.001) while non-significant decreases were observed in the MPC and CHV groups; the magnitude of changes significantly differed between groups (P < 0.001). Change in average monthly medication costs [for all conditions] also differed significantly between groups. The CHV + CCT group had a significant increase in participants reporting monthly medication costs (13.6%, CI: 7.2,19.9%; P < 0.001) whereas only nominal changes were observed in the MPC and CHV groups (three group change comparison *P* = 0.005). For diabetes medications, small and non-significant increases in the proportion of participants with expenditures were observed in the MPC and CHV + CCT groups, while a significantly smaller proportion of CHV participants (− 18.7%, CI: − 30.2,-7.2%; *P* = 0.001) reported monthly diabetes medication costs at endline relative to baseline (three group change comparison P = 0.001). The proportion of participants incurring any out-of-pocket payments for blood glucose monitoring supplies increased significantly in all groups (39.2% CHV + CCT, 16.1% MPC, 13.5% CHV) but the magnitude of change across groups was similar (*P* = 0.137).

At the household level, total health expenditures in the past month were similar in all groups (baseline *P* = 0.209; endline *P* = 0.173) and did not significantly change. Asset sales to pay for health in the preceding 3 months were relatively uncommon (25–30%) and similar in all intervention groups at baseline (*P* = 0.132), but significantly decreased among MPC recipients by 5.2% (CI: − 9.6,-0.8%; *P* = 0.019) at endline; asset sale changes were not significant in the CHV only (*P* = 0.651) or CHV + CCT (*P* = 0.101) groups, nor were differences in change across groups (*P* = 0.732). Borrowing to pay for health expenses in the preceding 3 months was more common (75–80%) and similar between groups at baseline, with statistically significant decreases (− 12.5 to − 26.1%, *P* < 0.05 in all groups) of similar magnitudes (*P* = 0.876) in all groups over the study period.

### Health service utilization

**Fig. 3:**
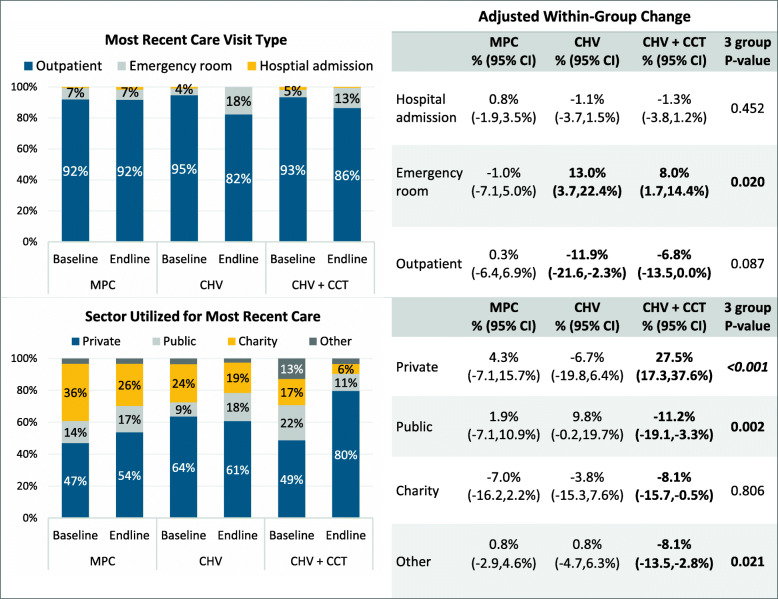
Baseline, Endline, and Adjusted Change in Health Facility Utilization by Group

Health service utilization was assessed in terms of self-reported regular care-seeking, the most recent diabetes care visit, and utilization of specific provider types in the preceding 6 months. Baseline and endline descriptive group comparisons are provided in Additional File 2 and Fig. 3; unadjusted and adjusted change are presented in Table 4. Self-reported regular doctors’ visits for diabetes care was significantly lower among CHV only participants at baseline relative to other groups, while at endline significantly more CHV + CCT participants reported regular care compared to those in the CHV only and MPC groups. Changes in self-reported regular doctors’ visits for diabetes care were statistically significant between groups (*P* = 0.008) with regular care visits decreasing, albeit not significantly, among MPC and CHV participants, and increasing significantly among CHV + CCT participants (15.1%, CI: 5.4,24.8%; *P* = 0.002). There were no significant changes or differences in change between groups in the proportion of participants attributing lack of care-seeking to cost.

**Table 4:** Change Diabetes Care Utilization and in Clinical Measurements

	UNADJUSTED CHANGE							ADJUSTED CHANGE ^**a**^						
	MPC		CHV only		CHV + CCT		3 groupcomp.*P*-value	MPC		CHV only		CHV + CCT		3 group comp. P-value
	%	(95% CI)	%	(95% CI)	%	(95% CI)		%	(95% CI)	%	(95% CI)	%	(95% CI)	
**Diabetes Care Utilization**														
Regular diabetes care visits	−7.1%	(−16.3,2.1)	−2.8%	(− 13.4,7.8)	**15.3%**	**(5.9,24.7)**	**0.002**	−4.9%	(−14.5,4.7)	−0.5%	(−11.9,10.9)	**15.1%**	**(5.4,24.8)**	**0.008**
Regular care not sought b/c of cost	−4.4%	(−12.5,3.6)	−3.0%	(−9.1,3.0)	2.2%	(−0.9,5.3)	0.138	−5.4%	(−13.6,2.8)	− 3.4%	(− 10.3,3.6)	2.1%	(− 1.6,5.7)	0.140
**Health Provider Visits** ^**b**^														
General Practitioner	2.2%	(−6.2,10.5)	3.5%	(−7.8,14.8)	**8.3%**	**(0.6,15.9)**	0.548	0.9%	(−7.8,9.6)	1.7%	(−10.0,13.3)	*7.9%*	*(−0.1,15.9)*	0.425
Other doctor/specialist	2.7%	(−8.9,14.4)	***−25.6%***	***(−38.7,-12.6)***	***19.2%***	***(9.5,28.8)***	***< 0.001***	1.8%	(−10.3,13.9)	***−27.8%***	***(−41.5,-14.0)***	**16.8%**	**(6.6,27.0)**	***< 0.001***
Pharmacist	−6.4%	(−16.3,3.6)	***−24.1%***	***(−37.5,-10.7)***	−11.2%	(−20.2,-2.2)	0.112	−6.1%	(−16.1,4.0)	**−24.1%**	**(−37.9,-10.4)**	**− 12.7%**	**(− 22.2,-3.2)**	0.106
Hospital Visit	−2.4%	(−11.5,6.8)	− 11.1%	(− 22.1,-0.1)	−0.9%	(−9.1,7.3)	0.321	−3.3%	(− 12.9,6.2)	**− 11.5%**	**(−22.9,-0.1)**	−1.6%	(− 10.6,7.3)	0.359
Provider selected for cost-related reasons	−5.4%	(−15.7,4.9)	0.3%	(− 14.0,14.7)	***− 25.5%***	***(− 35.4,-15.7)***	**0.003**	− 4.1%	(−14.9,6.7)	1.9%	(−12.8,16.7)	***−22.8%***	***(− 33.5,-12.2)***	**0.007**
Did not receive all needed care due to cost ^c^	−2.9%	(−14.6,8.8)	−5.0%	(− 19.2,9.2)	***− 25.2%***	***(− 35.3,-15.2)***	**0.008**	−3.6%	(− 15.6,8.4)	−6.4%	(− 21.5,8.7)	***−26.2%***	***(− 36.7,-15.7)***	**0.009**
**Body Mass Index (BMI)**														
BMI (mean)	**− 0.7**	**(−1.4,-0.1)**	− 0.4	(− 1.1,0.3)	**− 0.8**	**(−1.4,-0.1)**	0.698	− 0.6	(− 1.3,0.0)	−0.7	(− 1.5,0.1)	**−1.0**	**(− 1.7,-0.3)**	0.728
Normal (BMI < 25 kg/m^2^)	0.9%	(− 1.1,2.8)	3.0%	(− 1.6,7.6)	0.7%	(−2.5,3.8)	0.681	0.6%	(−1.6,2.8)	3.2%	(−1.5,7.9)	0.9%	(−2.7,4.5)	0.607
Overweight (BMI 25–29 kg/m^2^)	**7.0%**	**(1.4,12.5)**	0.4%	(−6.6,7.4)	−0.6%	(−6.8,5.6)	0.150	4.5%	(−1.4,10.4)	−1.2%	(−8.5,6.1)	− 1.8%	(− 8.5,5.0)	0.281
Obese (BMI > 30 kg/m^2^)	**−7.9%**	**(− 13.5,-2.3)**	−3.4%	(−10.2,3.4)	0.0%	(− 5.9,5.8)	0.163	− 5.1%	(− 11.0,0.8)	−2.0%	(− 8.8,4.8)	0.9%	(− 5.5,7.3)	0.365
**Blood Glucose**														
HbA1C (mean %)	−0.2	(−0.4,0.0)	***− 0.6***	***(− 0.8,-0.3)***	**−0.4**	**(− 0.6,− 0.2)**	0.073	**-0.2**	**(− 0.5,0.0)**	***−0.7***	***(− 1.1,-0.4)***	***− 0.5***	***(− 0.7,-0.3)***	**0.032**
HbA1C < 7.0%	4.2%	(− 2.4,10.8)	6.0%	(− 2.1,14.1)	**9.2%**	**(2.3,16.1)**	0.582	5.4%	(−1.5,12.2)	**9.1%**	**(0.1,18.0)**	**11.7%**	**(4.4,19.1)**	0.427
HbA1C = 7.0–7.9%	0.4%	(−7.0,7.9)	1.1%	(−6.5,8.7)	−2.0%	(− 9.0,5.1)	0.827	0.1%	(−7.6,7.8)	0.3%	(−7.8,8.5)	−2.8%	(−9.9,4.4)	0.806
HbA1C ≥ 8.0%	−4.7%	(−11.1,1.8)	−7.1%	(−15.3,1.2)	**−7.3%**	**(− 13.7,-0.9)**	0.831	−5.4%	(− 12.1,1.3)	**−9.4%**	**(− 18.7,-0.1)**	**− 9.0%**	**(− 15.8,-2.1)**	0.682
**Blood Pressure (BP)**														
Systolic blood pressure (mean)	−2.6	(−5.9,0.7)	−1.3	(−4.6,2.0)	− 1.3	(− 4.0,1.3)	0.816	− 2.3	(− 5.7,1.1)	−0.9	(−4.4,2.6)	− 0.4	(−3.3,2.5)	0.676
Diastolic blood pressure (mean)	**− 1.8**	**(−3.6,0.0)**	**−2.9**	**(− 5.2,-0.6)**	***− 3.7***	***(− 5.5,-1.8)***	0.354	−1.6	(− 3.5,0.3)	**− 2.5**	**(− 5.0,0.0)**	**−3.3**	**(− 5.3,-1.3)**	0.463
Normal BP (< 140/90)	**8.5%**	**(0.2,16.7)**	5.8%	(−3.4,15.1)	**12.0%**	**(4.3,19.8)**	0.588	8.4%	(−0.1,16.8)	6.2%	(−3.9,16.2)	**11.3%**	**(3.2,19.4)**	0.704
High BP (> 140/90)	**−8.5%**	**(−16.7,-0.2)**	−5.8%	(−15.1,3.4)	**− 12.0%**	**(− 19.8,-4.3)**	0.588	− 8.4%	(− 16.8,0.1)	−6.2%	(− 16.2,3.9)	**−11.3%**	**(− 19.4,-3.2)**	0.704

Bold italic indicates statistically significant (*P* < 0.001) findings; bold indicates statistically significant (*P* < 0.05) findings; italic indicates statistically significant (*P* < 0.10) findings. *P*-values are for three group comparison of magnitude of change during the study period. ^a^ Adjusted analyses controlled for participant sex, age, education level, and marital status; household size and the presence of more than one UNHCR registration case; prior diagnoses of hypertension, other chronic NCD diagnoses, and years since initial diabetes diagnosis; total household expenditure in the prior month; and receipt of humanitarian assistance, specifically the total value of cash assistance received in the prior month, and current WFP beneficiary status/transfer modality (voucher or Choice); ^b^ % with ≥1 visit in the past 6 months; ^c^ at most recent care visit

Changes in cost-motivated provider selection and not receiving all needed care because of cost were significant only among participants in the CHV + CCT group. The proportion of participants selecting their provider for cost-related reasons was similar between groups at baseline but significantly higher among MPC and CHV only participants compared to CHV + CCT participants at endline following a significant decrease of − 22.8% (CI: − 33.5,-12.2%; *P* < 0.001) in the CHV + CCT group (magnitude of changes in other groups was not significant; significant difference in change between groups, *P* = 0.007). At baseline, significantly more CHV + CCT participants reported not receiving all recommended care/services due to cost compared to the CHV only and MPC groups (*P* = 0.004). Proportions reporting inability to afford all recommended care decreased in all groups over the study period, though change was significant only in the CHV + CCT group (26.2%, CI: − 36.7,-15.7%; *P* < 0.001; difference in change between groups *P* = 0.009).

Regarding visits in the preceding 6 months by type of health provider, no significant changes were observed among MPC recipients. CHV + CCT participants reporting at least one visit to a general practitioner increased marginally over the study period by 7.9% (CI: − 0.1,15.9%; *P* = 0.051), but changes were similar in all three groups (*P* = 0.425). The most notable difference was in specialists visits where approximately half of participants in all groups reported specialist care at baseline whereas at endline, specialist care was significantly higher among CHV + CCT participants (66.1%) compared to CHV only (26.6%) and MPC participants (47.1%) (*P* < 0.001). Specialist visits significantly increased among CHV + CCT participants (16.8%, CI: 6.6,27.0%; *P* = 0.001), but decreased in CHV only participants (− 27.8%, CI: − 41.5,-14.0%; *P* < 0.001) (group difference in change *P* < 0.001). Pharmacist consultation and hospital visits were similar across groups at baseline and endline and similarly decreased in all groups. Pharmacist consultation decreased significantly in the CHV only (− 24.1%, CI: − 37.9,-10.4%; *P* = 0.001) and CHV + CCT groups (− 12.7%, CI: − 22.2,-3.2%; *P* = 0.009), whereas decreased hospital visits were significant only among CHV only participants (− 11.5%, CI: − 22.9,-0.1%; *P* = 0.049).

While all groups reported similar visit types for their most recent diabetes care, emergency room and outpatient visits significantly changed in the CHV only and CHV + CCT groups (Fig. 3). In the CHV only group, emergency room visits increased by 13.0% (CI: 3.7,22.4%; *P* = 0.006) while the CHV + CCT group had a lesser increase of 8.0% (CI: 1.7,14.4%; *P* = 0.013). Although emergency room visit change was significantly different between groups (*P* = 0.020), group changes in outpatient visits (*P* = 0.087) and hospital admissions (*P* = 0.452) were similar.

Diabetes care was most recently received at a private sector facility for most participants, with fewer seeking care in the charity and public sectors (Fig. 3). Statistically significant changes in sector were observed only in CHV + CCT participants, with an increase private sector utilization (27.5%, CI: 17.3,37.6%; *P* < 0.001) and corresponding decreases in other sectors (public sector: -11.2%, CI: − 19.1,-3.3%, *P* = 0.005; charity facilities: -8.1%, CI: − 15.7,-0.5%; *P* = 0.037; and other facilities: -8.1%, CI: − 13.5,-2.8%; *P* = 0.003). Changes significantly differed between groups for utilization in the private sector (*P* < 0.001), public sector (*P* = 0.002), and other facility types/sectors (*P* = 0.021), but not charity facilities (*P* = 0.806).

### Medication adherence, self-monitoring, and risk factors

**Fig. 4:**
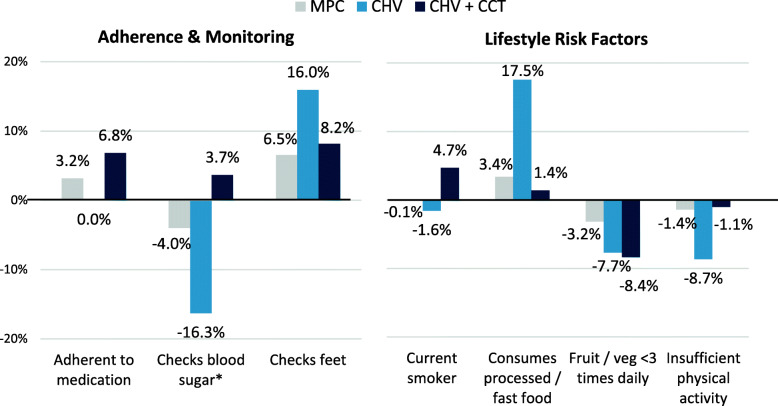
Adjusted Change in Medication Adherence, Self-Monitoring, and Lifestyle Risk Factors. *Legend:* * Statistically significant difference (*P* < 0.001) in change between groups. Bold text indicates statistically significant (*P* < 0.05) change within respective group

Figure 4 shows adjusted changes in medication adherence, self-monitoring, and lifestyle risk factors for each intervention group. Medication adherence was similarly high in all groups at baseline (> 88%, *P* = 0.267; based on Adherence to Refills and Medications Scale for Diabetes [ARMS-D] cutoff of 24) [30]. Significant increases in adherence were observed only in the CHV + CCT group (6.8%, CI: 2.2,11.5%; *P* = 0.004). While CHV + CCT participants had significantly higher medication adherence at endline (*P* = 0.036), changes in the MPC (*P* = 0.214) and CHV only (*P* = 0.995) groups were not statistically significant, nor was the difference in change between groups (*P* = 0.120).

Blood glucose self-monitoring was significantly higher among CHV + CCT (89.2%) and CHV only (88.3%) participants than MPC recipients (80.6%) at baseline. At endline, self-monitoring was highest in the CHV + CCT group (93.9%) with significantly fewer CHV only and MPC participants reporting self-monitoring (72.7 and 76.0%, respectively; *P* < 0.001). Change in self-monitoring was significant among CHV only participants (− 16.3%, CI: − 25.2,-7.4%; P = < 0.001), but not for MPC (*P* = 0.308) and CHV + CCT (*P* = 0.197) participants; the magnitude of change differed significantly different between groups (*P* = 0.001). In contrast, similar proportions in all three groups reported ever checking their feet at baseline (*P* = 0.637), whereas at endline, significantly more CHV only (91.1%) and CHV + CCT (85.7%) participants reported foot checking compared to MPC recipients (79.2%). Changes did not significantly differ between groups (*P* = 0.213), though foot checking did increase significantly in both the CHV only (16.0%, CI: 7.7,24.2%; *P* < 0.001) and CHV + CCT groups (8.2%, CI: 0.4,15.9%; *P* = 0.040).

Regarding lifestyle behaviors, smoking was significantly higher among CHV only and CHV + CCT participants both at baseline (27.6 and 26.1%, respectively; *P* = 0.020) and endline (25.8 and 30.7%, respectively; *P* = 0.007); however, no significant changes within each group nor difference in change across groups were observed. Consumption of processed and/or fast food in a typical week was similarly reported by all groups at baseline (*P* = 0.747), but increased significantly among CHV only participants (17.5%, CI: 6.3,28.7%; *P* = 0.002) with marginally significant differences in change between groups (*P* = 0.062). The proportion of participants consuming at least three servings of fruit and/or vegetables daily was also similar between groups at baseline (*P* = 0.385) and endline (*P* = 0.182), though significant decreases were observed in the CHV only (− 7.7%, CI: − 14.5,-0.9%; *P* = 0.027) and CHV + CCT groups (− 8.4%, CI: − 15.0,-1.7%; *P* = 0.014); MPC group change (*P* = 0.329) and group differences (*P* = 0.481) in change were not statistically significant. Finally, significantly more MPC recipients did not meet the World Health Organization’s (WHO) recommendations on physical activity (defined as < 150 min of moderate-intensity activity per week, or equivalent [31]) at baseline (51.7%) and endline (51.4%) compared to CHV only (42.9 and 35.2%) and CHV + CCT (33.5 and 32.4%) participants. Within-group changes and group differences in change in insufficient activity were not statistically significant.

### Clinical measurements

**Fig. 5:**
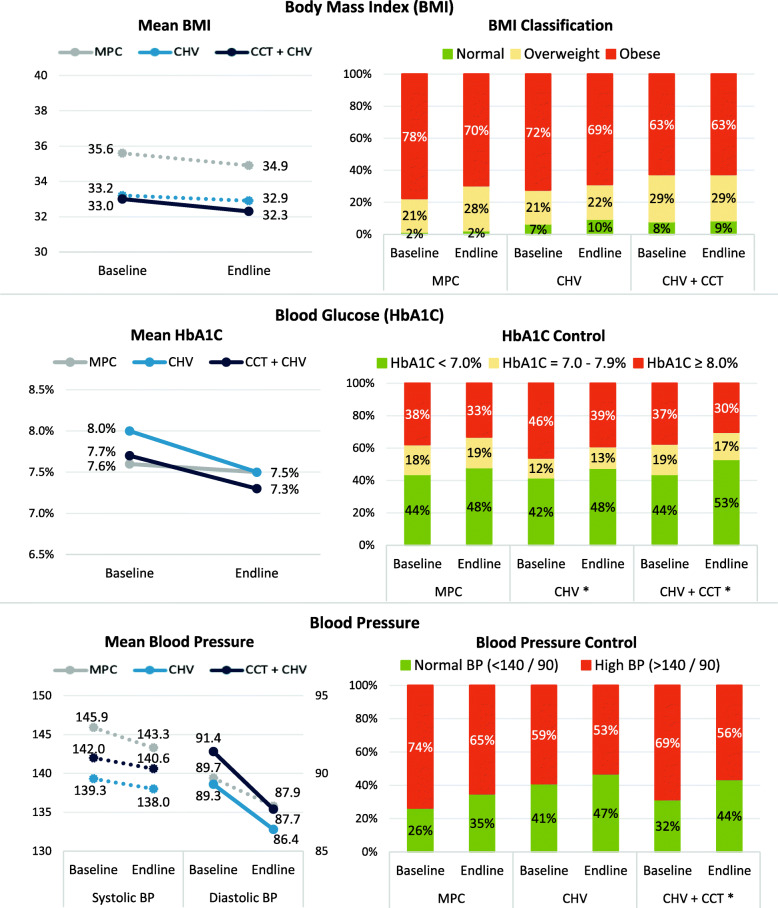
Clinical Measurements at Baseline and Endline by Intervention Group. *Legend:* Solid line indicates statistically significant adjusted change within group.* Indicates statistically significant adjusted change within group

Clinical outcomes were assessed using BMI, HbA1C, and blood pressure. Descriptive analysis of clinical measurements at baseline and endline are provided in Fig. 5 and Additional File 3; unadjusted and adjusted change over the study period are presented in Table 4. BMI was significantly higher among MPC recipients compared to CHV only and CHV + CCT participants both at baseline (*P* < 0.001) and endline (*P* = 0.001). While BMI decreased similarly in all groups (*P* = 0.728), change was only statistically significant in the CHV + CCT group (− 1.0 kg/m^2^, CI: − 1.7,-0.3; *P* = 0.005). Despite changes in average BMI, no significant changes in BMI classification (i.e., normal, overweight, obese) were observed.

HbA1C testing was used to assess blood glucose levels and, in turn, diabetes control. Average HbA1C levels were similar across groups at baseline and endline, but significantly decreased in all groups over time. The CHV only group had the largest change in average HbA1C, decreasing 0.7% (CI: − 1.1,-0.4%; P < 0.001) compared to − 0.5% (CI: − 0.7,-0.3%; P < 0.001) in the CHV + CCT group and − 0.2% (CI: − 0.5,0.0%; *P* = 0.028) in the MPC group (three group change comparison *P* = 0.032). Changes in average HbA1C did not significantly change glucose control classification of MPC recipients; however, the proportion of CHV only participants with HbA1C ≥ 8.0% decreased 9.4% (CI: − 18.7,-0.1%; *P* = 0.048) and those with HbA1C < 7.0% increased 9.1% (CI: 0.1,18.0%; *P* = 0.047). Among CHV + CCT participants, those with HbA1C ≥ 8.0% decreased 9.0% (CI: − 15.8,-2.1%; *P* = 0.011) and the proportion with HbA1C < 7.0% increased 11.7% (CI: 4.4,19.1%; *P* = 0.002). All changes in glucose control classification were similar between groups.

Average blood pressure also decreased in all groups, though decreases in systolic blood pressure were not statistically significant. Average diastolic blood pressure readings decreased − 3.3 mmHg (CI: − 5.3,-1.3; *P* = 0.001) among CHV + CCT participants and − 2.5 mmHg (CI: -5.0,0.0; P = 0.048) among CHV only participants; change in MPC recipients was not statistically significant (*P* = 0.092) and changes were similar between groups (*P* = 0.463). While changes are statistically significant, the magnitude of the changes are small and may not have clinical significance. The proportion of participants with “normal” blood pressure readings (classified as < 140/90 mmHg) increased similarly in all three groups (*P* = 0.704), though within-group change was only significant in the CHV + CCT group, where 11.3% (CI: 3.2,19.4%; *P* = 0.007) more participants had normal blood pressure readings at endline relative to baseline.

## Discussion

Changes in health expenditures for the CHV + CCT group consistently differed from the CHV only and MPC groups. Expenditures at the most recent outpatient diabetes care visit decreased from baseline to endline in all comparison groups, but the magnitude of change was smaller in the CHV + CCT group; declines in health facility payment amounts are likely a reflection of the April 2019 MoH policy changes that reduced user fees for Syrian refugees [12]. The CHV + CCT group was the only group with significant increases in the proportion of participants with health facility payments and in facility payment amounts for the most recent diabetes care visit in adjusted models. CHV + CCT beneficiaries were also the only group to report a significant rise in care-seeking at private health facilities (+ 27.5%), which is likely a contributing factor to increased health facility expenditures. Similarly, the CHV + CCT group was the only group with significant increases in the proportion of individuals with monthly medication expenditures and in expenditure amounts for all medication, compared to significant decreases in monthly diabetes medication costs in the CHV only group. Moreover, while blood glucose monitoring expenses increased significantly in all groups, the magnitude of increase was significantly greater in the CHV + CCT group. Taken together, these findings indicate that the combined conditional cash and education intervention (CHV + CCT group) was more successful than unconditional cash (MPC group) or education (CHV only) alone in raising diabetes-related expenditures.

The CCT + CHV group outperformed both the MPC and CHV only groups in health service utilization change over the one-year study period. In adjusted models, the CHV + CCT group was the only group with a significant increase in regular doctor visits (15.1%) and receipt of specialist care (16.7%) for diabetes. Interestingly, there were significant increases in emergency care in both the CHV only and CHV + CCT groups, which may be a result of greater awareness of their condition and concerning symptoms, however, overall hospital admissions (not necessarily related to complications of diabetes) remained constant among all the groups. The CHV + CCT group was also the only group where a significant decline in the proportion of patients not receiving all needed care due to cost was observed (− 26.2%) and where patients selected their provider due to cost-related reasons (− 22.8%). While health education may have increased demand for services in the CHV group, cost remained a barrier to care-seeking; when this barrier was removed by the addition of CCT, beneficiaries were empowered to seek care. In MPC households, diabetes care may not have been prioritized and previous evidence suggests that transfers are mostly spent on common goods (e.g., food and rent), though health expense are also frequently reported [32, 33]. In contrast, individuals receiving CCTs are intrinsically more able to spend the money on diabetes care because transfers are restricted to individual use for targeted conditions (i.e., requiring evidence of doctor visit or prescription) and, thus, presumably encourage spending on diabetes care.

Findings on diabetes self-care measures including medication adherence, blood glucose monitoring, and foot checks were mixed but suggest that both CCTs and health education were beneficial. Increases in the CHV and CHV + CCT groups indicate that the education component of the CHV intervention appears to have successfully increased foot checks among participants. Results for blood glucose monitoring were varied, where the CHV group saw declines and the CHV + CCT group had a significantly higher monitoring rate at endline compared to the other groups; it is possible that testing material costs were barriers to routine glucose checking and that the CHV group was most impacted as the only group not receiving any cash transfer. As the CHV + CCT group had the highest adherence of all groups at endline and was the only group for which medication adherence significantly improved during the study period, conditional transfers appear to have a positive impact on medication adherence, potentially by removing or reducing financial barriers to maintaining a regular supply of diabetes medication. This interpretation is supported by the higher endline expenditures for glucose monitoring supplies and diabetes medication in the CHV + CCT group.

No significant change in smoking or physical activity were observed during the one-year follow up period for any group, which is aligned with findings from other studies that have observed no, or modest, impacts of behavior change programs for NCDs among Syrian refugees [17, 34, 35]. Successful behavior change depends upon the combination of an individual’s intentions, skills, and psychosocial variables (e.g., attitudes, perceived norms, and self-efficacy concerning the behavior), in addition to the presence of environmental barriers to performing the behavior; thus, achieving change in behavioral risk factors often depends upon tailoring interventions to the desired behavior, target population, and context [36]. The absence of significant impacts of the current study interventions on smoking and physical activity likely reflect the multifaceted components necessary for effective behavior change.

With respect to diet, all groups saw decreases in fresh fruit and vegetable consumption of similar magnitude over the study period, which could be a reflection of the increasingly difficult financial situation faced by refugees that has translated to lower levels of household food security and adoption of a less diverse diet. According to WFP’s Comprehensive Food Security and Vulnerability Assessment/Monitoring Exercise, while food consumption has remained relatively consistent over time among Syrian refugees living in Jordanian host communities, increased use of livelihood-based coping strategies and an increasing proportion of household expenditures on food have led to deteriorations in food security where 20% of households were classified as food secure in 2018 compared to 28% in 2016 and 53% in 2014 [37–39]. Dietary diversity has also slightly declined; 78% of households had optimal dietary diversity scores in 2014 and 72% in 2016 versus 69% in 2018 [37–39]. The CHV intervention was the only group that reported a significant increase in consumption of processed foods; as with blood glucose monitoring, it is possible the observed declines relate to households’ poor economic situations, and that in CHV-only houses where cash is not received, processed foods may be favored for their lower purchase costs.

The general improving trend observed in blood glucose levels across groups is an encouraging finding with respect to diabetes control among Syrian refugees in Jordan, particularly given the potential lack of awareness among refugees of recent MoH policy changes that were intended to improve health access via reduced user fees. Mean HbA1C levels decreased significantly over time in all groups, with the largest declines in the CHV only and CHV + CCT groups, both of which had statistically significant increases in the proportion of patients with well controlled diabetes, suggesting that education was an important component in improving diabetes control. While larger increases in medication expenditures and adherence were observed in the CHV + CCT group, these did not translate to greater improvements in diabetes control, suggesting that cash may be beneficial for intermediate outcomes but that health education interventions are equally as effective in improving diabetes control.

### Limitations

Despite efforts for rigorous design and implementation of this study, like all research, results should be taken with consideration of certain limitations. CHV programs for NCDs as evaluated in low- and middle-income countries differ in their comprehensiveness, frequency of home visits, and linkage to clinical care; the CHV program assessed in this research was focused more minimally on education and quarterly visits for basic monitoring and individualized counseling. Additionally, WFP expanded its Choice program in Jordan during the study period and while participant-reported receipt of cash assistance from WFP did not substantially change over the course of our study, it is possible that more participants began receiving a choice in how to use cash from WFP. Revision of UNHCR targeting criteria for MPC recipients during the study period resulted in changes in intervention receipt for some study households, whereby 29 beneficiaries enrolled in the MPC group stopped receiving MPC from UNHCR while 73 participants in the CHV only and CHV + CCT groups not receiving MPC at baseline were added to the MPC beneficiary list. This diluted pure implementation of the planned interventions for all participants, though efforts were made to account for receipt of assistance outside the studied interventions, including UNHCR MPC, in adjusted analyses. Another important change was made in April 2019 to the Government of Jordan’s policy determining public sector healthcare costs for Syrian refugees. This likely influenced endline care utilization and made changes in public sector health expenditures more challenging to interpret than if costs were consistent during all reference periods. Additionally, the quality of expenditure data may be limited due to recall bias from self-report (as with many other outcomes) and difficulty accurately capturing spending across a range of categories. Moreover, expenditure by itself does not necessarily constitute an improvement and depends on the appropriateness of what it is being spent on, affordability, and whether other measures could have been taken to overcome need to spend, which were not captured in this study. The lack of a true control group receiving no additional interventions potentially weakened our ability to draw conclusions about direct causality between a single intervention and outcomes. While efforts were made in sampling, intervention group allocation, and analysis to account for receipt of assistance outside the studied interventions, it is likely that some participants received additional assistance, whether cash transfers or through another modality, during our study. Despite efforts to coordinate cash transfer receipt by vulnerability levels and across organizations, there were differences between beneficiary groups and it is also possible there was selection bias; while the adjusted models attempt to control observed differences, it is possible that some differences or factors beyond the interventions influenced study outcomes. Finally, the study was limited to greater Amman and Zarqa, representing largely urban refugees, and is not representative of Syrian refugees in Jordan more broadly; thus, results may not be generalizable to rural or unregistered refugee populations or areas where health service access substantially differs.

## Conclusion

Findings from this study indicate that conditional cash and health education and health education alone were equally successful at improving diabetes control, but that conditional cash combined with education had additional benefits for blood pressure and intermediate measures including medication adherence and diabetes-related expenditures. The CHV + CCT group was the only group to consistently see significant improvement in clinical outcomes, including decreased mean BMI and HbA1C as well as proportions of participants with poor diabetes control and high blood pressure. The CHV only group also saw significant decreases in mean HbA1C levels and the proportion of participants with poor diabetes control, whereas the MPC group had significant decreases in mean HbA1C that were smaller in magnitude and did not translate to improved disease control classification. Results indicate that unconditional cash, specifically household-level MPC, alone are insufficient to improve chronic disease outcomes and where there are specific health objectives, individual-level conditional transfers may be preferable, in particular when coupled with health education where cost is a barrier to accessing care. Encouragingly, the community health education intervention was almost as effective alone as when combined with conditional cash. When considering cash transfers and NCDs, humanitarian agencies implementing large-scale unconditional cash transfer programs should consider targeted top-ups for individuals with chronic diseases to reduce financial access barriers to medication and care. Robust monitoring could allow further assessment of the impact of CCT top-ups on health access, service utilization, and disease control Organizations working in the health sector should continue community health education interventions, as these may be equally as effective as cash transfers, and where possible, provide conditional cash or coordinate with others providing cash transfers to maximize benefits, where conditional cash and health education combined are an ideal approach if resources are available to support a more comprehensive intervention.

## Supplementary Information

**Additional file 1.** Overview and Description of Outcome Measures. Descriptions of main outcome measures analyzed.

**Additional file 2.** Care Utilization and Costs Incurred for Diabetes Maintenance in Jordan by Intervention Group at Baseline and Endline. Descriptive analyses of care utilization and health expenditure outcomes by group at baseline and endline.

**Additional file 3.** Participant Biometrics by Intervention Group at Baseline and Endline. Descriptive analyses of participant biometrics at baseline and endline.

## Data Availability

The datasets supporting the conclusions of this article are available in the Humanitarian Data Exchange, and [upon acceptance] can be accessed at https://data.humdata.org/dataset/cct-chv-mpc-syrian-refugees-jordan.

## Abbreviations

ARMS-D: Adherence to Refills and Medications Scale for Diabetes
BMI: Body mass index
BP: Blood pressure
CCT: Conditional cash transfers
CHV: Community health volunteer
CI: 95% confidence interval
HbA1C: Glycated hemoglobin
HH: Household
JOD: Jordanian Dinar
MoH: Ministry of Health
MPC: Multi-purpose cash
NCD: Noncommunicable disease
NGO: Non-governmental organization
SD: Standard deviation
UNHCR: United Nations High Commissioner for Refugees
US$: U.S. Dollars
VAF: Vulnerability Assessment Framework
WFP: World Food Programme
WHO: World Health Organization

## Footnotes

1 Multiple UNHCR registration cases primarily occurred when there was more than one traditionally defined family in a household according to the study’s definition of a household (people who share a living space, meals, and financial resources).
