# Supplementary material for: Improving diabetes control for Syrian refugees in Jordan: a longitudinal cohort study comparing the effects of cash transfers and health education interventions
Source: Confl Health. 2021 May 25;15:41. doi: 10.1186/s13031-021-00380-7 (PMC8145855; doi:10.1186/s13031-021-00380-7)
Supplement: Supplementary file 1 — Additional file 1. Overview and Description of Outcome Measures. Descriptions of main outcome measures analyzed. [file 13031_2021_380_MOESM1_ESM.pdf]

Additional File 1. Overview of Outcome Measures

Health Expenditures for Diabetes Care and Maintenance

Outcome Measure	Description
**Health Facility Payments for Most Recent Outpatient Diabetes Care**	
% with any payment at most recent care visit	Within the past year
Total paid at facility for most recent care visit	Includes consultation fees, diagnostic testing, and medications obtained at health facility during the most recent visit to health facility, hospital outpatient department, or emergency room (without overnight stay)
**Average Monthly Medication Costs**	
% with any monthly medication costs for all conditions	In average month
Average monthly medication costs for all conditions	Measured among all participants
% with any monthly medication costs for diabetes	
Average monthly medication costs for diabetes	
**Blood Glucose Monitoring Supplies**	
% with any monitoring supply costs	In average month
Average monthly costs for monitoring supplies	Either at home or in a pharmacy
**Routine Spending on Health**	
Total household health expenditures	In the past month<br>Includes expenditures for consultation, diagnostic testing, medication, and associated transportation
% of participants who sold assets to pay for health	In the past three months
% of participants who borrowed money to pay for health	In the past three months

Diabetes Care Utilization Measures

Outcome Measure	Description
% able to receive regular diabetes care	"Regular" defined as at least one care visit every six months
% not regularly seeking diabetes car because of cost	
% with ≥1 visit to select types of providers	In the past six months
Most recent care visit type	Type of most recent care visit within the past year
Sector utilized for most recent care	For most recent care visit within the past year
% who selected provider for cost-related reasons	For most recent care visit (within the past year)
% who did not receive all needed care due to cost	At most recent care visit (within the past year)

Medication Adherence, Self-Monitoring, and Lifestyle Risk Factor Measures

Outcome Measure	Description
% adherent to medication (per ARMS-D)	Binary variable generated from composite scores of the 12-item ARMS-D scale, which measures the ability to take and refill medications in various circumstances self-reported for each item on a scale representing “none of the time”, “most of the time”, “some of the time”, and “all of the time”. Scale cutoff for adherence vs. non-adherence is 24.
% who check blood sugar	Participants who reported ever checking their blood sugar (at home or in a pharmacy)
% who check feet	Participants who reported ever checking their feet
% current smokers	Participants who reported being current smokers
% consuming processed/fast food	Based on reported consumption in a typical week in the past year
% consuming fruit/vegetables <3 times daily	Based on reported consumption in a typical week in the past year
% with insufficient physical activity	Based on reported activity in a typical week in the past year

Clinical Outcome Measures

Outcome Measure	Description
Mean Body Mass Index (BMI)<br>BMI category classification	 Normal: BMI < 25 kg/m2<br>Overweight: BMI 25 - 29 kg/m2<br>Obese: BMI > 30kg/m2
Mean HbA1C %<br>Blood glucose control/HbA1C classification	 HbA1C < 7.0%<br>HbA1C = 7.0 - 7.9%<br>HbA1C ≥ 8.0%
Mean systolic blood pressure<br>Mean diastolic blood pressure<br>Blood pressure category classification	 <br>Normal BP: <140/90<br>High BP: >140/90

Analysis of all indicators included measures at baseline and endline
