# Supplementary material for: Improving diabetes control for Syrian refugees in Jordan: a longitudinal cohort study comparing the effects of cash transfers and health education interventions
Source: Confl Health. 2021 May 25;15:41. doi: 10.1186/s13031-021-00380-7 (PMC8145855; doi:10.1186/s13031-021-00380-7)
Supplement: Supplementary file 2 — Additional file 2. Care Utilization and Costs Incurred for Diabetes Maintenance in Jordan by Intervention Group at Baseline and Endline. Descriptive analyses of care utilization and health expenditure outcomes by group at baseline and endline. [file 13031_2021_380_MOESM2_ESM.pdf]

Care Utilization and Costs Incurred for Diabetes Maintenance in Jordan by Intervention Group at Baseline and Endline

		BASELINE				ENDLINE			
		MPC (N=201) Point (95% CI)	CHV only (N=156) Point (95% CI)	CHV+CCT (N=203) Point (95% CI)	P-value	MPC (N=175) Point (95% CI)	CHV only (N=128) Point (95% CI)	CHV+CCT (N=179) Point (95% CI)	P-value
**Out-of-Pocket Costs (in US$)**									
**Costs Incurred for Most Recent Care Visit**									
**Health Facility Payments for Outpatient Care[1]**		*n=150*	*n=112*	*n=161*		*n=119*	*n=79*	*n=176*	
Any payment for outpatient care at facility (%)		64.0% (56.2-71.8%)	73.2% (64.9-81.5%)	74.5% (67.7-81.3%)	0.095	***62.2% (53.3-71.0%)***	***67.1% (56.5-77.7%)***	***88.1% (83.2-92.9%)***	***<0.001***
Total paid at facility for visit[2]	Median	11 --	14 --	14 --		9 --	9 --	17 --	
	Mean	25.9 (19.8-31.9)	35.9 (27.0-44.8)	30.7 (23.3-38.0)	0.191	**15.4 (11.3-19.5)**	**19.6 (11.6-27.7)**	**28.2 (22.3-34.1)**	**0.006**
**Average Monthly Medication Costs**		*n=201*	*n=156*	*n=203*		*n=175*	*n=128*	*n=179*	
**Medication for All Conditions**									
Any monthly medication costs (%)		82.6% (77.3-87.9%)	86.5% (81.1-92.0%)	79.8% (74.2-85.4%)	0.247	**84.0% (78.5-89.5%)**	**88.3% (82.6-93.9%)**	**95.5% (92.5-98.6%)**	**0.002**
Average monthly medication costs[2]	Median	28 --	32 --	28 --		28 --	28 --	55 --	
	Mean	35.8 (30.9-40.7)	43.8 (37.2-50.5)	36.3 (31.2-41.4)	0.093	***37.1 (31.2-43.0)***	***43.5 (33.4-53.7)***	***59.8 (53.0-66.6)***	***<0.001***
**Medication for Diabetes**									
Any monthly diabetes medication costs (%)		**52.7% (45.8-59.7%)**	**69.2% (61.9-76.6%)**	**66.5% (60.0-73.1%)**	**0.002**	***56.6% (49.2-64.0%)***	***53.1% (44.4-61.9%)***	***76.5% (70.3-82.8%)***	***<0.001***
Average monthly diabetes medication costs[2]	Median	4 --	11 --	9 --		4 --	4 --	13 --	
	Mean	**9.4 (7.2-11.5)**	**15.3 (12.0-18.6)**	**12.2 (10.0-14.3)**	**0.006**	***11.3 (8.3-14.2)***	***11.2 (6.7-15.8)***	***20.1 (16.5-23.6)***	***<0.001***
**Blood Sugar Monitoring Supplies[3]**									
Any payments for blood sugar monitoring supplies		20.9% (15.2-26.6%)	21.8% (15.2-28.3%)	29.1% (22.8-35.4%)	0.116	***36.0% (28.8-43.2%)***	***36.7% (28.3-45.2%)***	***69.8% (63.0-76.6%)***	***<0.001***
Average monthly monitoring supply costs[2]	Median	0	0 --	0 --		0 --	0 --	14 --	
	Mean	3.7 --	4.3 (2.6-6.1)	4.9 (3.5-6.3)	0.490	***7.0 (5.2-8.8)***	***6.5 (4.6-8.5)***	***20.2 (16.7-23.8)***	***<0.001***
**Routine Spending on Health**									
Health Expenditures (past month)[4]	Median	85 --	85 --	96 --		93 --	124 --	147 --	
	Mean	111.8 (98.2-125.5)	122.8 (95.0-150.6)	142.3 (111.2-173.4)	0.209	154.8 (111.2-198.5)	188.0 (142.8-233.2)	214 (166.2-261.0)	0.173
Sold assets to pay for health (past 3 months; %)		10.9% (6.6-15.3%)	17.3% (11.3-23.3%)	17.2% (12.0-22.5%)	0.132	**6.3% (2.7-9.9%)**	**16.4% (9.9-22.9%)**	**12.3% (7.4-17.1%)**	**0.019**
Borrowed to pay for health (past 3 months; %)		47.8% (40.8-54.7%)	51.9% (44.0-59.9%)	50.7% (43.8-57.7%)	0.712	36.6% (29.4-43.8%)	39.8% (31.2-48.4%)	38.0% (30.8-45.2%)	0.845
**Care Utilization**									
Reports regular doctor visits for diabetes care (%)		**56.2% (49.3-63.1%)**	**44.2% (36.4-52.1%)**	**55.7% (48.8-62.6%)**	**0.045**	***49.1% (41.7-56.6%)***	***41.4% (32.8-50.1%)***	***70.9% (64.2-77.7%)***	***<0.001***
Regular care not received because of cost (%)		94.3% (89.4-99.3%)	97.7% (94.5-100.9%)	97.8% (94.7-100.9%)	0.350	**89.9% (83.5-96.3%)**	**94.7% (89.5-99.9%)**	**100% --**	**0.049**
**Visits to Health Providers in the past 6 months (% with ≥1 visit)**									
General Practitioner		82.1% (75.9-88.3%)	78.8% (71.1-86.4%)	79.9% (73.7-86.1%)	0.777	84.3% (77.7-90.9%)	82.3% (73.7-90.9%)	88.1% (83.3-92.9%)	0.406
Other doctor/specialist		44.4% (36.4-52.4%)	52.2% (42.9-61.6%)	47.0% (39.2-54.7%)	0.445	***47.1% (38.1-56.1%)***	***26.6% (16.6-36.5%)***	***66.1% (59.1-73.1%)***	***<0.001***
Pharmacist		70.9% (63.5-78.2%)	82.3% (75.2-89.4%)	75.6% (69.0-82.3%)	0.100	64.5% (55.8-73.1%)	58.2% (47.1-69.3%)	64.4% (57.3-71.5%)	0.597
Hospital Visit		27.2% (20.0-34.3%)	30.1% (21.5-38.7%)	27.4% (20.5-34.3%)	0.851	24.8% (17.0-32.6%)	19.0% (10.1-27.8%)	26.6% (20.0-33.1%)	0.425
Provider selected for cost-related reasons (%)		58.3% (50.3-66.2%)	47.8% (38.4-57.1%)	55.5% (47.8-63.2%)	0.225	***52.9% (43.9-61.9%)***	***48.1% (36.8-59.4%)***	***29.9% (23.1-36.8%)***	***<0.001***
Did not receive all needed care at most recent visit due to cost (%)		**41.7% (33.8-49.7%)**	**44.2% (34.9-53.5%)**	**59.1% (51.5-66.7%)**	**0.004**	38.8% (30.0-47.7%)	39.2% (28.2-50.2%)	33.9% (26.9-40.9%)	0.590

Exchange rate: 1 JOD = US$ 1.41. Bold italic indicates statistically significant (P < 0.001) findings; Bold indicates statistically significant (P < 0.05) findings; Italic indicates statistically significant (P < 0.10) findings.

[1] Includes consultation fees, diagnostic testing, and medications obtained at health facility during the most recent visit to health facility, hospital outpatient department, or emergency room (without overnight stay); [2] Among all participants; [3] At home or in a pharmacy; [4] Includes expenditures for consultation, diagnostic testing, medication, and associated transportation.
