# Supplementary material for: Improving diabetes control for Syrian refugees in Jordan: a longitudinal cohort study comparing the effects of cash transfers and health education interventions
Source: Confl Health. 2021 May 25;15:41. doi: 10.1186/s13031-021-00380-7 (PMC8145855; doi:10.1186/s13031-021-00380-7)
Supplement: Supplementary file 3 — Additional file 3. Participant Biometrics by Intervention Group at Baseline and Endline. Descriptive analyses of participant biometrics at baseline and endline. [file 13031_2021_380_MOESM3_ESM.pdf]

Participant Biometrics by Intervention Group at Baseline and Endline

		BASELINE							ENDLINE						
		**MPC (N=201)**		**CHV only (N=156)**		**CHV+CCT (N=203)**			**MPC (N=175)**		**CHV only (N=128)**		**CHV+CCT (N=179)**		
		Point	(95% CI)	Point	(95% CI)	Point	(95% CI)	*P* -value	Point	(95% CI)	Point	(95% CI)	Point	(95% CI)	*P* -value
**Body M<ass Index (BMI)**		*n=200*		*n=151*		n=201			*n=168*		*n=125*		n=174		
BMI	Median	34.4		33		31.8			34		33		31		
	Mean	***35.6***	***(34.6-36.6)***	***33.2***	***(32.3-34.2)***	***33.0***	***(32.1-34.0)***	***<0.001***	**34.9**	**(33.8-36.0)**	**32.9**	**(31.8-33.9)**	**32.3**	**(31.4-33.2)**	**0.001**
Normal (BMI < 25 kg/m^2)		**1.5%**	**(-0.2-3.2%)**	**6.6%**	**(2.6-10.6%)**	**8.0%**	**(4.2-11.7%)**	**0.004**	**2.4%**	**(0.1-4.7%)**	**9.6%**	**(4.4-14.8%)**	**8.6%**	**(4.4-12.8%)**	**0.048**
Overweight (BMI 25 - 29 kg/m^2)		**21.0%**	**(15.3-26.7%)**	**21.2%**	**(14.6-27.8%)**	**29.4%**	**(23.0-35.7%)**		**28.0%**	**(21.1-34.8%)**	**21.6%**	**(14.3-28.9%)**	**28.7%**	**(21.9-35.5%)**	
Obese (BMI > 30 kg/m^2)		**77.5%**	**(71.7-83.3%)**	**72.2%**	**(65.0-79.4%)**	**62.7%**	**(55.9-69.4%)**		**69.6%**	**(62.6-76.7%)**	**68.8%**	**(60.6-77.0%)**	**62.6%**	**(55.4-69.9%)**	
**Blood Glucose**		*n=201*		*n=156*		*n=203*			*n=175*		*n=128*		*n=179*		
HbA1C	Median	7.3		7.6		7.3			7		7		7		
	Mean	7.6	(7.4- 7.9)	8.0	(7.7- 8.3)	7.7	(7.4- 8.0)	0.173	7.5	(7.2- 7.8)	7.5	(7.2- 7.8)	7.3	(7.0- 7.6)	0.638
HbA1C < 7.0%		43.8%	(36.9-50.7%)	41.7%	(33.8-49.5%)	43.8%	(37.0-50.7%)	0.297	48.0%	(40.5-55.5%)	47.7%	(38.9-56.4%)	53.1%	(45.7-60.5%)	0.436
HbA1C = 7.0 - 7.9%		18.4%	(13.0-23.8%)	12.2%	(7.0-17.4%)	18.7%	(13.3-24.1%)		18.9%	(13.0-24.7%)	13.3%	(7.3-19.2%)	16.8%	(11.2-22.3%)	
HbA1C ≥ 8.0%		37.8%	(31.0-44.6%)	46.2%	(38.2-54.1%)	37.4%	(30.7-44.2%)		33.1%	(26.1-40.2%)	39.1%	(30.5-47.6%)	30.2%	(23.4-37.0%)	
**Blood Pressure**		*n=200*		*n=155*		*n=203*			*n=175*		*n=128*		*n=179*		
Systolic blood pressure	Median	145		136		139			140		137		136		
	Mean	**145.9**	**(143.1-148.7)**	**139.3**	**(135.7-143.0)**	**142.0**	**(139.0-145.0)**	**0.014**	143.3	(140.1-146.4)	138.0	(134.1-141.9)	140.6	(137.4-143.8)	0.108
Diastolic blood pressure	Median	90		88		92			88		86		88		
	Mean	89.7	(88.2-91.2)	89.3	(87.4-91.3)	91.4	(89.7-93.1)	0.208	87.9	(86.2-89.6)	86.4	(84.4-88.5)	87.7	(85.8-89.7)	0.532
Normal BP (<140/90)		**26.4%**	**(20.2-32.5%)**	**41.0%**	**(33.2-48.8%)**	**31.5%**	**(25.1-38.0%)**	**0.013**	34.9%	(27.7-42.0%)	46.9%	(38.1-55.6%)	43.6%	(36.2-50.9%)	0.081
High BP (>140/90)		**73.6%**	**(67.5-79.8%)**	**59.0%**	**(51.2-66.8%)**	**68.5%**	**(62.0-74.9%)**		65.1%	(58.0-72.3%)	53.1%	(44.4-61.9%)	56.4%	(49.1-63.8%)	

Bold italic indicates statistically significant (P < 0.001) findings; Bold indicates statistically significant (P < 0.05) findings; Italic indicates statistically significant (P < 0.10) findings.
